# Differential Leaf Age-Dependent Thermal Plasticity in the Keystone Seagrass *Posidonia oceanica*

**DOI:** 10.3389/fpls.2019.01556

**Published:** 2019-11-29

**Authors:** Miriam Ruocco, Pasquale De Luca, Lázaro Marín-Guirao, Gabriele Procaccini

**Affiliations:** ^1^Integrative Marine Ecology Department, Stazione Zoologica Anton Dohrn, Naples, Italy; ^2^Research Infrastructures for Marine Biological Resources Department, Stazione Zoologica Anton Dohrn, Naples, Italy; ^3^Seagrass Ecology Group, Oceanographic Center of Murcia, Spanish Institute of Oceanography, San Pedro del Pinatar, Spain

**Keywords:** seagrass, gene expression, photo-physiology, thermal plasticity, leaf age, DNA methylation

## Abstract

**Introduction:** Gene-expression patterns and their upstream regulatory mechanisms (e.g. epigenetic) are known to modulate plant acclimatability and thus tolerance to heat stress. Within species, thermal plasticity (i.e. temperature-sensitive phenotypic plasticity) and differential thermo-tolerance are recognized among different genotypes, development stages, organs or tissues. Leaf age and lifespan have been demonstrated to strongly affect photosynthetic thermo-tolerance in terrestrial species, whereas there is no information available for marine plants.

**Materials and Methods:** Here, we investigated how an intense warming event affects molecular and photo-physiological functions in the large-sized seagrass *Posidonia oceanica*, at fine spatial resolution. Plants were exposed for one week at 34°C in a controlled-mesocosm system. Subsequent variations in the expression of 12 target genes and global DNA methylation level were evaluated in three leaf-age sections (i.e. basal, medium and high) established along the longitudinal axis of youngest, young and fully mature leaves of the shoot. Targeted genes were involved in photosynthesis, chlorophyll biosynthesis, energy dissipation mechanisms, stress response and programmed cell death. Molecular analyses paralleled the assessment of pigment content and photosynthetic performance of the same leaf segments, as well as of plant growth inhibition under acute warming.

**Results:** Our data revealed, for the first time, the presence of variable leaf age-dependent stress-induced epigenetic and gene-expression changes in seagrasses, underlying photo-physiological and growth responses to heat stress. An investment in protective responses and growth arrest was observed in immature tissues; while mature leaf sections displayed a higher ability to offset gene down-regulation, possibly through the involvement of DNA methylation changes, although heat-induced damages were visible at photo-physiological level.

**Discussion:** Overall, mature and young leaf tissues exhibited different strategies to withstand heat stress and thus a variable thermal plasticity. This should be taken in consideration when addressing seagrass response to warming and other stressors, especially in large-sized species, where sharp age differences are present within and among leaves, and other gradients of environmental factors (e.g. light) could be at play. Molecular and physiological evaluations conducted only on adult leaf tissues, as common practice in seagrass research, could give inadequate estimates of the overall plant state, and should not be considered as a proxy for the whole shoot.

## Introduction

The ability to constantly sense and adjust to environmental challenges is fundamental for all organisms to maintain their homeostasis, but it is especially important for plants, because of their sessile lifestyle. The plant stress response changes among and within species, due to their inherent biological and ecological attributes, and according to characteristics of the stressor, such as intensity and duration (Buchanan et al., 2015). At the organismal level, such differences can be associated to the stage of development or to genotype-specific features of individual plants and can occur among organs or tissues, down to the single cell types (Buchanan et al., 2015). The vast heterogeneity and complexity of the stress response is often undervalued, and this may lead to misleading inferences and oversimplifications of the effects of environmental stressors on plants' acclimation capacity and ultimately survival.

In the context of ongoing climate changes, assessing the thermal plasticity (i.e. temperature-sensitive phenotypic plasticity) and differential thermo-tolerance of plants at different levels of biological organization, is particularly relevant. Most research in this field has been conducted in terrestrial higher plants, mainly in economically and dietary important crops, where high temperatures are major threats for agricultural production and food safety (Röth et al., 2015). Heat stress adversely affects plant growth, development, physiological processes and yield (reviewed in Hasanuzzaman et al., 2013). Variability in thermal tolerance has been observed among different genotypes (e.g. Rainey and Griffiths, 2005; Koscielny et al., 2018) and life stages (Wahid et al., 2007; Zhou et al., 2017), as well as within plant organs (e.g. leaves; Yamada et al., 1996; Kalituho et al., 2003; Zhang et al., 2012; Marias et al., 2017a; Marias et al., 2017b).

In particular, leaf age and lifespan are known to strongly influence plant photosynthetic thermo-tolerance (Yamada et al., 1996; Zhang et al., 2012). In *Coffea arabica*, chlorophyll fluorescence-based analysis has showed that mature leaves have a significantly greater T_crit_ (i.e. the temperature at which the basal fluorescence F_0_ rise began on F_0_-T curves) than younger leaves, and this was considered as an evolutionary adaptation for protecting older leaves from irreversible damage (Marias et al., 2017b). Similarly, Zhang et al. (2012) found that leaf lifespan was positively correlated to T_crit_, suggesting that longer leaf persistence is associated to greater thermo-tolerance of the photosynthetic apparatus. Besides age differences across leaves, in monocotyledonous plant species, a strong vertical age gradient is also present within leaves. Along the longitudinal axis, undifferentiated cells are placed at the base of the leaf blade, while the most mature cells occur at the leaf tip (Evert et al., 1996). This gradient of leaf developmental stages is produced by coordinated and localized transitions in gene-expression patterns, resulting in different biochemical and physiological compartments occurring along the leaf length (e.g. Li et al., 2010; Li et al., 2015; Mattiello et al., 2015). Each leaf segment is also responsible for variable photosynthetic capacity (Evert et al., 1996), and thus photosynthetic thermo-tolerance may possibly change across leaf-age sections. Spatial heterogeneity of chlorophyll-fluorescence parameters during stress events (e.g. drought or osmotic stress), underlying the patterns and gradients of photosynthesis, has been already demonstrated in plant leaves by means of fluorescence imaging techniques (e.g. Chaerle and Van Der Straeten, 2001; Massacci et al., 2008; Sperdouli et al., 2012; Li et al., 2015).

In marine plants (i.e. seagrasses), knowledge about the factors influencing heat-stress response and thermo-tolerance is much limited, despite predictions have challenged their long-term persistence under climate warming (Jordà et al., 2012), and extreme summer temperatures have already caused abrupt deterioration of seagrass populations (Díaz-Almela et al., 2009; Marbà and Duarte, 2010; Fraser et al., 2014; Thomson et al., 2014). Contrasting thermo-tolerance at photo-physiological and molecular levels has been found between individuals collected across latitudinal and depth gradients (e.g. Bergmann et al., 2010; Franssen et al., 2011; Winters et al., 2011; Marín-Guirao et al., 2017; Tutar et al., 2017; Marín-Guirao et al., 2018), or among plant developmental stages (e.g. Olsen et al., 2012; Salo and Pedersen, 2014; Guerrero-Meseguer et al., 2017; Hernán et al., 2017). However, these studies have been conducted considering the averaged response of the whole seagrass shoot or only mature leaf tissue (generally the middle section of second or third-rank leaves). Fine-resolution studies aimed at understanding the differential response to heat stress (or other abiotic stressors) within organs/tissues, for example in relation to leaf age, are currently completely missing in seagrasses. Especially in large-sized seagrasses, sharp age gradients are present both among and within the different leaves of the shoot (Alcoverro et al., 1998). This is exacerbated in canopy-forming species, where the strong light-attenuation gradient from the top to the meadow bottom parallels the vertical leaf-age gradient, thus influencing molecular and photo-physiological patterns across the leaf (e.g. Alcoverro et al., 1998; Dalla Via et al., 1998; Enríquez et al., 2002; Ralph et al., 2005; Marín-Guirao et al., 2015; Ruocco et al., 2019).

At molecular level, the processes of sensing and responding to heat stress in plants comprise the activation of numerous regulatory and signaling pathways that eventually lead to metabolic adjustments to minimize the damage and re-establish the cellular homeostasis (Kotak et al., 2007; Qu et al., 2013; Ohama et al., 2017). Heat-stress responsive genes/proteins include signaling components that regulate gene-expression responses and functional components that directly protect plant cells (Qu et al., 2013). In addition, an extensive reprogramming of primary metabolism occurs in response to heat stress (Allakhverdiev et al., 2008; Wang et al., 2018), where photosynthesis is regarded as one of the most heat-sensitive pathways, and is often inhibited before other cellular functions (Berry and Bjorkman, 1980). The regulation of gene expression under heat stress is often achieved by means of epigenetic mechanisms, including DNA methylation, histone modifications/variants, chromatin remodeling and small/long non-coding RNAs (Liu et al., 2015). First evidences that some of these processes are at play also in seagrasses in response to warming (Marín-Guirao et al., 2017) and other environmental cues (Greco et al., 2012; Greco et al., 2013), have been provided in recent years. In *Posidonia oceanica*, experimental temperature increase triggered the induction of genes involved in DNA and histone methylation, a potential way to favor an efficient heat acclimation through the activation and regulation of heat-responsive genes (Marín-Guirao et al., 2017; Marín-Guirao et al., 2019). In addition, DNA methylation changes across leaf-age sections have been recently demonstrated in the same species (Ruocco et al., 2019); however, if and eventually how, epigenetic mechanisms can contribute to stress acclimation processes of specific leaf-age sections remains to be assessed.

The main goal of this study was to investigate how thermal plasticity at molecular and photo-physiological levels and thermo-tolerance can vary within the same plant organ (i.e. leaf), as a function of tissue age. To address this question, the large-sized/long-living seagrass *P. oceanica* was selected as target species, as a wide range of tissue ages is present within the shoots, with leaves reaching up to 300 days (Alcoverro et al., 1998); therefore, we hypothesize that the response to heat stress and thermo-tolerance can change across leaf-age sections. We exposed plants to a short-term (1-week) acute heat stress (34°C) in an indoor mesocosm system and determined the expression gradients of genes associated to key primary metabolic processes (photosynthesis, chlorophyll biosynthesis, mitochondrial respiration, general stress response and programmed cell death), in three sections (i.e. basal, medium and high) established along the longitudinal axis of three ranked leaves (i.e. leaf 1, 2 and 3) within the *P. oceanica* shoot. Shifts in target gene expression were correlated with chlorophyll fluorescence-derived photosynthetic parameters and pigment content of the same leaf segments. Fitness-related traits at the whole-plant level (i.e. relative leaf growth rate and necrotic surface) were also determined. Finally, to further investigate the role played by epigenetic mechanisms in the acclimative response of *P. oceanica* to heat stress and its interplay with leaf age, we estimated changes in the level of global DNA methylation (5-mC) on a subset of leaf-age segments under acute warming.

## Materials and Methods

### Plant Collection, Mesocosm System, and Experimental Design

In July 2016, large *P. oceanica* ramets consisting of an apical portion of horizontal rhizomes bearing numerous (ca. 15–20) connected vertical shoots, were randomly collected by SCUBA diving, from a shallow-water meadow (8–10 m depth) located around the island of Procida (Gulf of Naples, Italy 40°45.218'N, 14°01.400'E). Half of the experimental plants used in this study came from the experiment described in Ruocco et al. (2019). Seawater temperature (T) in the Gulf of Naples annually ranges between 13.82–28.96°C with an average T of 18.12°C. The temperature of 28.96°C was the maximum recorded in July 2017 (data from ARPAC DT. U.O. MARE, 2013–2018; http://www.arpacampania.it).

Plant material was transported to an indoor mesocosm facility at the Stazione Zoologica Anton Dohrn (Naples, Italy) in large coolers filled with ambient seawater within 1-2hr after collection. The experimental system has previously been described in details (Ruocco et al., 2019). Briefly, it consists of six independent aquaria (500 L) equipped with two LED lamps which allow the simulation of light diel fluctuation and natural light spectra at a given depth. Temperatures in the mesocosms were controlled by aquarium chiller/heaters (Teco TK 2000). The mesocosm circuits were filled with natural seawater obtained from a nearby-unpolluted area, and seawater quality was maintained through mechanical filtration and UV sterilization. Continuous light and temperature measurements were performed using a LI-COR LI-1400 quantum sensor and HOBO^®^ Pendant^®^ UA-002-64 data loggers (Onset Computer Corporation), respectively. Salinity was measured daily using a WTW Cond 3310 portable conductivity meter and kept along the experiment within the range of 37.3–37.7.

Twelve plant fragments of similar size and shoot number were carefully selected, and individually attached by clamps to twelve plastic net cages (34 × 24 × 10 cm) filled with coarse sediments. Two randomly selected cages were placed in each of six independent mesocosms. Large fragments of *P. oceanica* were preferred over small ones to ensure the optimal conditions of plants during the experimental period (Marín-Guirao et al., 2013) and to maintain the canopy structure responsible for regulating the light environment within the meadow (Sandoval-Gil et al., 2014).

**Figure 1:**
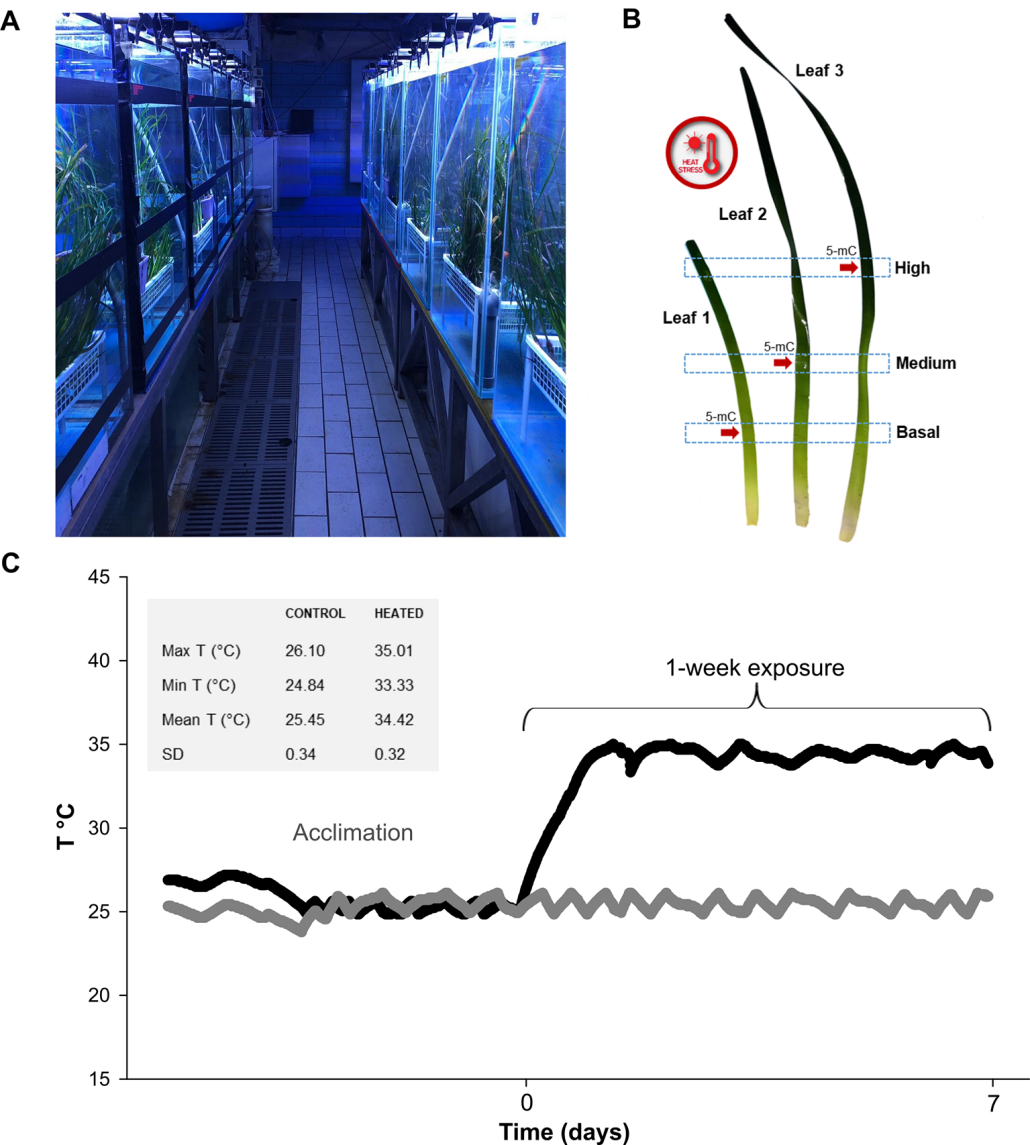
In **(A)** general view of the mesocosm system at Stazione Zoologica Anton Dohrn (Naples—Italy); **(B)** time course of temperature during acclimation and exposure phases, measured with HOBO data loggers every 10 min. Temperature values in control and exposure tanks are represented with gray and black symbols, respectively. Max, min, mean T (°C), and SD values recorded during the exposure phase are depicted in the gray box; **(C)** graphical depiction of leaf sections used for molecular and photo-physiological analyses. Global DNA methylation (5-mC) level was assessed on a sub-set of leaf sections (indicated with red arrows).

Before starting the experimental treatment, plants were acclimated in the aquaria for 1 week, under environmental conditions similar to those experienced by the natural population during the period in which plants were sampled: temperature = ca. 25°C; max. noon irradiance = ca. 400 µmol photons m^-2^ s^-1^ above the canopy; 14:10 h light:dark photoperiod). Subsequently, temperature in half of the tanks was rapidly increased to 34°C at a rate of 0.4°C h^-1^ (**Figure 1A**). The reason for choosing such an intense temperature ramping was to induce an acute heat stress in a short time period. In this way, leaf-age related differences in plant responses at molecular, photo-physiological and morphological levels, were likely to be amplified and sub-lethal stress biomarkers could be detected. The temperature value is within the highest extreme of summer seawater temperature ranges (+4–5°C) projected for the Mediterranean Sea by the end of the century (Sánchez et al., 2004; Jordà et al., 2012; IPCC, 2014). Yet, it could be rapidly reached in confined waters like coastal lagoons and intertidal ponds, where water temperatures are often naturally beyond the theoretical tolerance limit of seagrass species (Tomasello et al., 2009).

Plant exposure lasted one week (**Figure 1A**), after which samples were collected for the different analyses as follows. One randomly selected vertical shoot from each of the twelve pots was used to analyze variations in photosynthetic parameters, pigment content and gene expression. From each shoot, three leaves were selected, namely leaf 1 (youngest), leaf 2 (young) and leaf 3 (mature), and three sections were established along their longitudinal axis: B (basal)—the lowest portion at 5 cm distance from the ligule; M (medium)—the intermediate section at 20 cm distance from the ligule; H (high)—the upper section at 40 cm distance from the ligule (**Figure 1B**). A 3-cm tissue section above and below the established heigths, was collected. The newborn leaf of the shoot (< 3 cm) was discarded due to its small size, as were rank leaves 4 and 5, due to the high epiphytic cover, the presence of nectrotic marks and broken tips. Photo-physiological and gene-expression responses were determined on two shoots per tank (one per pot). Within each tank, all measurements were averaged, since we consider the tank as the true experimental replicate (n° of replicates used in statistical tests n = 3; tot. biological replicates N = 6). Global DNA methylation level in heated and control plants was assessed on a sub-set of aformentioned leaf sections (**Figure 1B**).

### Fitness-Related Traits

Relative leaf growth rate was determined using the Zieman method (Zieman, 1974), that is by marking the boundary limit between the leaf and the ligule with a fine needle. Shoots were marked right after the acclimation phase at the onset of the experimental treatment. They were subsequently collected at the end of the thermal exposure to estimate the surface area of newly formed tissue (below the mark) and thus to infer leaf growth. The relative growth rate was then estimated as the ratio of leaf material produced before marking to that produced after marking divided by the time interval and expressed as leaf surface produced by day (cm^2^ cm^-2^ day^-1^). The area of leaf necrotic tissues (brown necrotic tissue on the leaf surface or at the leaf tip) was also recorded and expressed as total necrotic surface (cm^2^ shoot^-1^). As for the other data, values obtained for the two pots within each tank were averaged to be used as independent replicates (n = 3).

### Photo-Physiology

A diving-PAM portable fluorometer (Walz, Germany) was used to characterize the functioning of the photosynthetic apparatus at the level of photosystem II (PSII). Chlorophyll *a* fluorescence measurements were performed as previously described in Marín-Guirao et al. (2013). Briefly, the saturation pulse method was used to measure the basal (F_0_) and maximum fluorescence (Fm) and to calculate the maximum photochemical efficiency of PSII [(Fv/Fm = (Fm-F_0_)/Fm] in the selected leaf segments of plants adapted to darkness overnight (i.e. before switching on the illumination system). The rapid light curve (RLC) method was subsequently applied on the same leaf segments at noon, after 5 hours under illumination in the aquaria, to obtain the relative electron transport rate (rETR), the effective quantum yield (ΔF/Fm') and non-photochemical quenching (NPQ). Non-photochemical quenching was calculated as NPQ = (Fm-Fm')/Fm' where Fm' is the maximum fluorescence of light-adapted leaves.

Following the chlorophyll *a* fluorescence measurements, the analyzed leaf segments (108 in total) were detached from the shoots and stored in complete darkness at -80°C for pigment analysis. Pigment extraction was carried out by homogenizing 1-cm^2^ leaf segments in 80% acetone, buffered with MgCO3 solution to prevent acidification of the extract (Dennison, 1990). Extracts were stored at 4°C in the dark for 24 h and subsequently centrifuged (1,000g for 10 min at 4°C). The absorbance of the extracts was then determined spectrophotometrically at 470, 646, 663, and 725 nm, using a 1 ml quartz-glass cuvettes. The chlorophyll *a* and *b* concentrations, as well as the total carotenoid concentration, were calculated using the equations defined by Lichtenthaler and Wellburn (1983) and expressed as µg cm^-2^.

### Sample Genotyping

Genomic DNA was extracted from about 30 mg dried leaf tissue collected from each individual plant employed for the experiment, using the NucleoSpin^®^ Plant II kit (Macherey-Nagel) following manufacturer's instructions. DNA quality was assessed through 1.0% agarose gel electrophoresis; DNA purity and concentration were determined using a NanoDrop^®^ ND-1000 Spectrophotometer (Thermo Fisher Scientific). Individual multi-locus genotypes were assessed by a total of 11 putatively neutral microsatellite loci (SSRs) in two separate multiplex PCR reactions consisting of a 5-plex and a 6-plex, in a 12.5-µL final reaction volume (see Jahnke et al., 2015 for further details). PCR products were analyzed on an Automated Capillary Electrophoresis Sequencer 3730 DNA Analyzer (Applied Biosystems) and electropherogram profiles were visualized using the software PeakScanner (Applied Biosystems). Identification of distinct multilocus genotypes was obtained with the software Gimlet (Valière 2002). The scoring analysis revealed that all but two ramets allocated in the distinct experimental tanks represented distinct multilocus genotypes (see **Table S1**).

### Reverse Transcription-Quantitative Polymerase Chain Reaction

RT-qPCR analysis was used to explore differences in expression levels of target genes in control *vs.* heated conditions among and within *P. oceanica* leaves. Leaf sub-samples were collected from the same shoots employed for pigment analysis (108 samples). Epiphyte-free material was entirely submerged in RNAlater^©^ tissue collection (Ambion, life technologies), stored one night at 4°C, and finally stowed at -20°C until RNA extraction. Total RNA was extracted with Aurum™ Total RNA Mini Kit (BIO-RAD) following manufacturer's instructions. RNA purity and concentration were checked using NanoDrop^®^ ND-1000 spectrophotometer (Thermo Fisher Scientific) and RNA quality was assessed through 1.0% (w/v) agarose 0.5× Tris/borate/ethylenediaminetetraacetic acid gel (0.5 mg/ml ethidium bromide) electrophoresis. Subsequently, total RNA (500 ng) from each sample was retro-transcribed into cDNA with the iScript™ cDNA synthesis kit (BIO-RAD), according to manufacturer's protocol. Twelve genes of interest (GOIs) were selected within functional categories potentially affected by heat stress, namely photosynthesis, general cellular stress response (CSR), mitochondrial respiration and programmed cell death (PCD) (Hasanuzzaman et al., 2013). Specifically, a number of genes involved in light reaction functions of photosynthesis (e.g. photosystem subunits and electron carriers) and carbon fixation, were targeted (*psbA*, *psbD*, *psbC*, *PSBS*, *FD*, and *RBCS*). Since heat stress is known to reduce chlorophyll accumulation (Mathur et al., 2014), a chlorophyll *a-b* binding proteins (*CAB-151*), and a key enzyme involved in the chlorophyll biosynthetic pathways (*POR*), were also selected. General stress-responsive genes, such as heat shock proteins (*HSP90* and *SHSP*), a negative regulator of PCD (*BI*), and a key gene involved in mitochondrial energy dissipation mechanisms (*AOX*), were also targeted. For full gene and protein names, see **Table 1**. Two reference genes (*18S* and *L23*) were selected and used for target gene-expression normalization on the basis of previous works conducted in the same species under heat stress and other abiotic stressors (Serra et al., 2012; Dattolo et al., 2014; Lauritano et al., 2015; Marín-Guirao et al., 2016). All primer sequences were already available in the literature (see **Table 1**).

**Table 1:** List of genes of interest assessed in *P. oceanica* using reverse transcription-quantitative polymerase chain reactions.

Gene	Protein	*E*	R^2^	Reference
psbA	Photosystem II protein D1	92%	0.99	Dattolo et al., 2014
psbD	Photosystem II protein D2	100%	0.98	Dattolo et al., 2014
psbC	Photosystem II CP43 reaction center protein	93%	0.99	Ruocco et al., 2019
PSBS	Photosystem II 22 kDa protein	100%	0.99	Dattolo et al., 2014
FD	Ferredoxin-1, chloroplastic	100%	0.98	Dattolo et al., 2014
RBCS	RuBisCO small subunit	100%	0.99	Dattolo et al., 2014
CAB-151	Chlorophyll *a-b* binding protein 151, chloroplastic	93%	0.99	Dattolo et al., 2014
POR	Protochlorophyllide reductase	98%	0.99	Ruocco et al., 2018
AOX	Alternative oxidase 1a	100%	0.99	Procaccini et al., 2017
BI	Bax inhibitor 1	100%	0.98	Traboni et al., 2018
HSP90	Heat shock protein 90	100%	0.99	Lauritano et al., 2015
SHSP	Small heat shock protein	99%	0.98	Lauritano et al., 2015

Gene and protein names, percent efficiency (E), correlation coefficient (R^2^), and references are given.

RT-qPCR reactions were performed in a Viia7 Real Time PCR System (Applied Biosystems) using Fast SYBR^®^ Green Master Mix (Applied Biosystems) as fluorescent detection chemistry. Reactions were carried out in a 10µl final volume with 5µl MM, 2µl of 1.4 pmol µl^-1^ primers and 3µl of 1:30 cDNA template and assembled in the 384-well plates format, by means of a Tecan Freedom EVO^®^ 200 automated liquid handling system. The thermal profile of the reactions was as follows: 95°C for 20 s, 40 times 95°C for 1 s and 60°C for 20 s. For determining the specificity of the reaction, the melting curve of each amplicon from 60 to 95°C was also detected. All RT-qPCR reactions were conducted in triplicate and each assay included three no-template negative controls. The technical variation among the triplicates was checked and individual outliers were excluded when SD was higher than 0.3. Primer's sequences, percent efficiencies (*E*) and regression coefficients (R^2^) of GOIs are reported in **Table 1**.

Relative quantification of gene expression was obtained following Livak and Schmittgen (2001). In details, the negative differences in cycles to cross the threshold value between the RGs and the respective GOI (-ΔCT) were calculated according to equation (1). Mean -ΔCT values were then calculated for biological replicates of each leaf rank (i.e. 1, 2 and 3), leaf height (i.e. basal, medium and high) and treatment (i.e. control and heated), from individual -ΔCT values. Data collected from the two *P. oceanica* fragments placed in each tank were averaged (*n* = 3). Fold expression changes were definitely obtained with the equation (2):

(1) $$-∆{\text{CT=CT}}_{\text{RGs}}-{\text{CT}}_{\text{GOI}}$$

(2) Fold expression change =+2(|(−ΔCT treatment)−(−ΔCT control )|)

### DNA Methylation (5-mC)

For evaluating the effect of heat stress on global genome methylation (5-mC), DNA was extracted from the following leaf sections: leaf 1—basal, leaf 2—medium, and leaf 3—high. One *P. oceanica* shoot per tank, in controls and heated conditions, was employed (*n* = 3). A 5-cm tissue section was accurately cleaned of epiphytes and desiccated with silica gel (Sigma-Aldrich). Genomic DNA was isolated using the same kit as for the sample genotyping (see above). DNA quality was assessed through 1.0% agarose gel electrophoresis, DNA purity was estimated using a NanoDrop^®^ ND-1000 Spectrophotometer (Thermo Fisher Scientific) and concentration was accurately determined by Qubit dsDNABR assay kit using the Qubit 2.0 Fluorometer (Thermo Fisher Scientific). Global DNA methylation was determined colorimetrically by an ELISA-like reaction with the MethylFlash™ Methylated DNA Quantification Kit (Epigentek Inc.), following manufacturer's instructions. Two technical replicates were conducted for each reaction, starting from 50 ng DNA per sample. Absorbance at 450 nm was assayed using a Multiskan™ FC Microplate Photometer (Thermo Fisher Scientific). Results are reported as % methylated DNA (5-mC) relative to the input DNA quantity for each leaf sample.

### Statistical Analysis

Multivariate statistics was used to assess the overall signal of all photo-physiological variables (photosynthetic parameters and pigment content) and 12 GOIs (using -ΔCT values). Specifically, a permutational multivariate analysis of variance (PERMANOVA) was conducted with the Primer 6 v.6.1.12 & PERMANOVA + v.1.0.2 software package (PRIMER-E Ltd) (Clarke and Gorley, 2006). The analysis consisted of three fixed factors: Leaf Rank (LR) with three levels (1, 2, and 3), Leaf Height (LH) with three levels (Basal, Medium, and High), and Treatment with two levels (Control and Heated). Following, a univariate three-way analysis of variance (ANOVA) was conducted to detect the effects of leaf rank, leaf height, and treatment on single photo-physiological variables and individual gene-expression values (-ΔCT values). Global DNA methylation data were analysed by two-way ANOVA with two fixed factors: Leaf Section (LS) with three levels and Treatment with two levels (Control and Heated). Differences in relative leaf growth rate and necrosis between control and heated plants were tested by a Student's *t*-test. Normality of data was tested using the Shapiro-Wilk test and variance homogeneity was verified using Levene's test. When parametric assumptions were not met, data were Box-Cox transformed. Student–Newman–Keuls post-hoc test was used whenever significant differences were detected. All ANOVAs were performed using the statistical package STATISTICA (StatSoft, Inc. v. 10). To facilitate the visualization of photosynthetic and pigment responses, mean values relative to controls (± propagated SE) were used for graphical representation (Winters et al., 2011), yet the statistical analyses were done with the raw data. Propagated SEs were calculated with an online error propagation calculator (https://www.eoas.ubc.ca/courses/eosc252/error-propagation-calculator-fj.htm).

Raw photo-physiological data from control and heated plants can be found in **Table S2**. A thorough interpretation of the differences among the selected leaf sections of control plants have been published previously in Ruocco et al. (2019).

## Results

### Fitness-Related Traits

**Figure 2:**
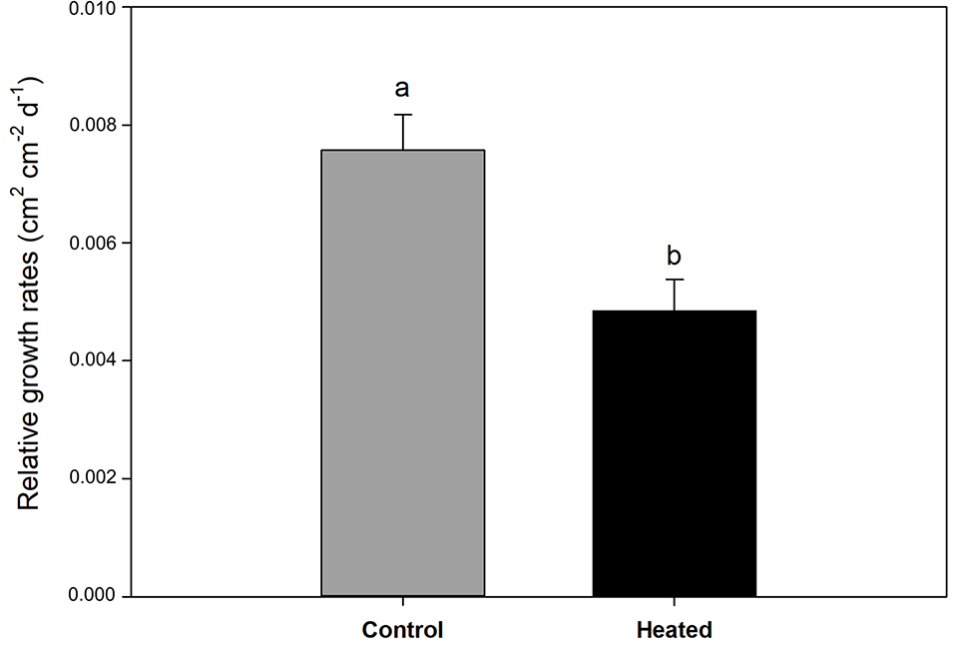
Relative leaf growth rate (cm^2^ cm^-2^ day^-1^) in control and heated *P. oceanica* plants. Different letters indicate significant differences at *P* < 0.05. Data are mean ± SE.

Relative leaf growth rate was 36% lower in *P. oceanica* plants exposed to acute heat stress, in comparison with controls (*t*-test; *P* < 0.05) (**Figure 2**). Experimental treatment did not significantly affect leaf necrotic surface (*t*-test; *P* = 0.07), although there was a tendency for plants exposed to 34°C to increase necrotized tissue (mean ± SE: control plants = 41.8 ± 7.09, heated plants = 59.3 ± 5.04 cm^2^ shoot^-1^).

### Photosynthetic Parameters and Pigment Content

**Figure 3:**
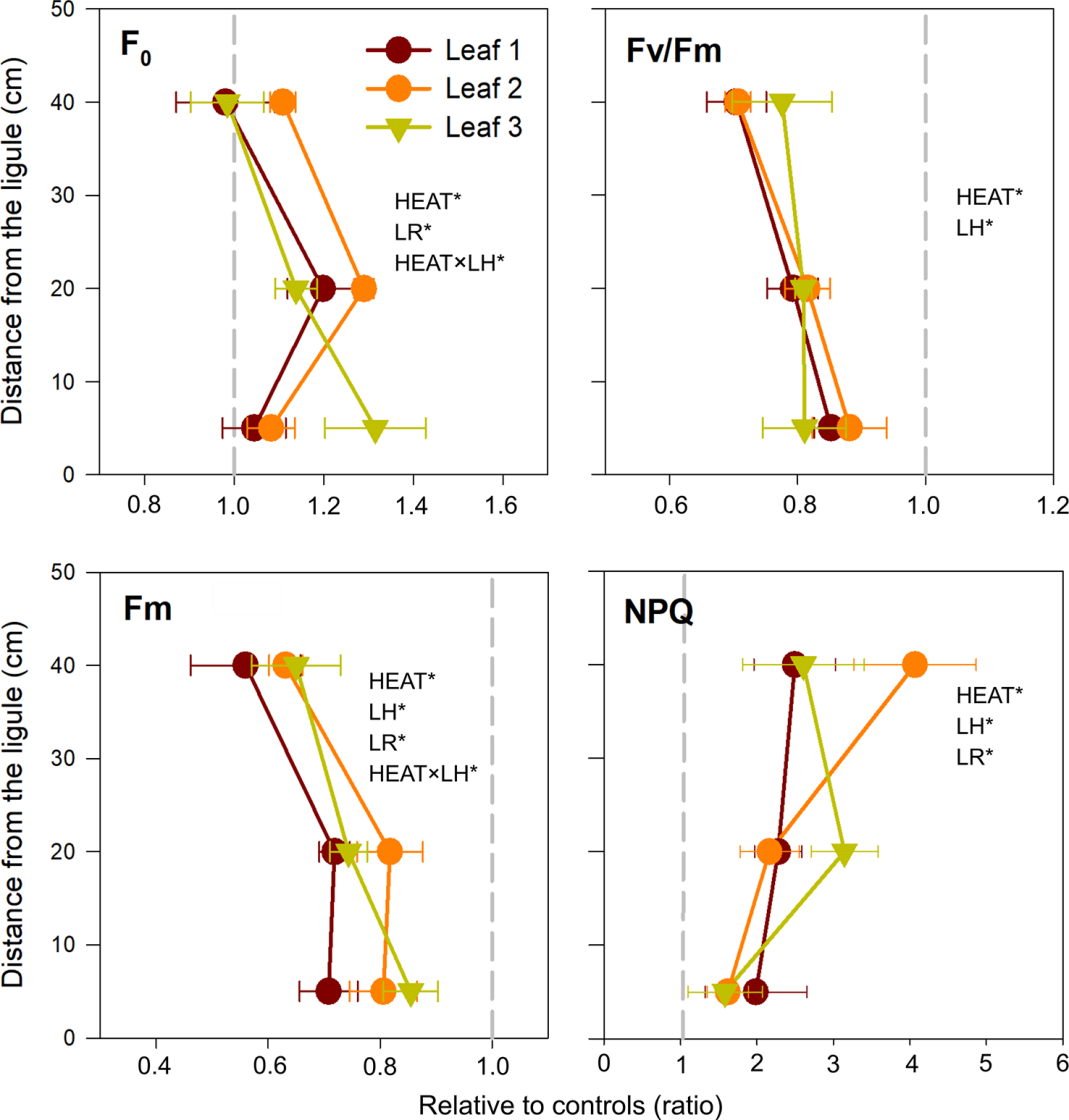
Changes in photosynthetic parameters, in heated relative to control plants (ratio). F_0_, basal fluorescence; Fv/Fm, maximum photochemical efficiency of PSII; Fm, maximal fluorescence; NPQ, non-photochemical quenching. Values on the left or right of the dashed gray line represent a decrease or increase in respect to controls, respectively. Data are mean ± SE (*n* = 3). Significant results of three-way analyses of variance are reported on the top of the graphs. For details of SNK post-hoc tests see **Table S3.**

Multivariate analysis (three-way PERMANOVA) of photo-physiological variables highlighted the distinct role of specific leaf portions in determining *P. oceanica* response to heat stress. Globally, photosynthetic parameters and pigment content were significantly affected by the factors LH [*P*_(perm)_ < 0.001], LR [*P*_(perm)_ < 0.001], and heat stress [*P*_(perm)_ < 0.001], and notably by the combination Heat × LH [*P*_(perm)_ < 0.01] (**Table 2**). Multivariate pairwise comparisons within each level of the factor LH emphasized a significant effect of intense warming on all selected leaf sections [control *vs.* heated in B: *P*_(MC)_ < 0.001, M: *P*_(MC)_ < 0.001 and H: *P*_(MC)_ < 0.001] (**Table 2**). Univariate three-way ANOVA confirmed that all analyzed chlorophyll *a* fluorescence-derived photosynthetic parameters were affected by heat stress and varied according to LH and/or LR, individually or in combination. Basal fluorescence (F_0_) was significantly higher in plants exposed to 34°C only in basal (SNK, *P* < 0.05) and middle leaf segments (SNK, *P* < 0.001) (**Tables 3** and **S3**; **Figure 3**), whereas maximum fluorescence (Fm) exhibited the largest decrease in apical leaf sections (SNK, *P* < 0.001) (**Tables 3** and **S3**; **Figure 3**).

**Table 2:** Results of three-way permutational multivariate analyses of variance conducted on photo-physiological variables (photosynthetic parameters and pigment content), and multivariate gene-expression data (-ΔCT values). *P*_(perm)_ < 0.05 are in bold.

**Three-Way Permutational Multivariate Analysis of Variance**					
**Source**	**df**	**Pseudo-F**	**P** **_(perm)_**	**Unique perms**	**Pair-wise tests**
*Photo-physiology*					
Heat	1	196.23	**0.000**	9851	
Leaf Rank (LR)	2	12.406	**0.000**	9958	LR: 1 ≠ 2 = 3
Leaf Height (LH)	2	18.242	**0.000**	9957	LH: B = M ≠ H
Heat × LR	2	1.421	0.257	9950	
Heat × LH	2	6.281	**0.004**	9960	B: Control ≠ Heated; M: Control ≠ Heated; H: Control ≠ Heated
LR × LH	4	1.393	0.266	9957	
Heat × LR × LH	4	0.475	0.756	9948	
*GOIs*					
Heat	1	81.799	**0.000**	9945	
Leaf Rank	2	0.467	0.753	9959	
Leaf Height	2	3.168	**0.019**	9941	LH: B ≠ H; M = B, H
Heat×LR	2	0.986	0.392	9954	
Heat×LH	2	0.510	0.717	9946	
LR×LH	4	0.662	0.699	9940	
Heat×LR×LH	4	0.779	0.595	9938	

Consequently, maximum quantum yield of PSII (Fv/Fm) largely decreased under heat stress (*P* < 0.001) regardless leaf rank and/or leaf height (**Table 3** and **Figure 3**). A similar pattern was observed for electron transport rate (r-ETR) and effective quantum yield (ΔF/Fm'), which were greatly depressed in heated plants, in all sections established along the leaf length (SNK, *P* < 0.001 for B, M, and H) (**Table S3**). NPQ was also significantly affected by the treatment (*P* < 0.001) (**Table 3**). It increased under acute heat stress, particularly in distal leaf sections (**Figure 3**).

**Table 3:** Results of three-way analyses of variance to assess the individual contribution of photo-physiological variables (photosynthetic parameters and pigment content) and GOIs.

**Three-Way Analysis of Variance**													
***Photo-physiology ***	**df**	**F** **_0_**		**Fm**		**Fv/Fm **		**NPQ**		**Chl ** ***a***		**Chl ** ***b***	
**Effect**		**F**	***P***	**F**	***P***	**F**	***P***	**F**	***P***	**F**	***P***	**F**	***P***
Heat	1	22.692	**0.000**	196.301	**0.000**	249.167	**0.000**	115.563	**0.000**	27.970	**0.000**	28.712	**0.000**
LR	2	24.530	**0.000**	12.412	**0.000**	3.053	0.060	5.785	**0.007**	1.350	0.272	0.661	0.523
LH	2	3.056	0.059	18.236	**0.000**	14.808	**0.000**	76.159	**0.000**	29.880	**0.000**	27.212	0.000
Heat×LR	2	1.095	0.345	1.421	0.255	0.537	0.589	0.305	0.739	2.050	0.144	0.417	0.662
Heat×LH	2	4.612	0.016	6.280	**0.005**	1.722	0.193	1.024	0.369	7.810	**0.002**	8.562	**0.001**
LR×LH	4	1.602	0.195	1.393	0.256	0.145	0.964	0.631	0.643	1.310	0.283	0.942	0.451
Heat×LR×LH	4	1.695	0.173	0.475	0.754	0.868	0.493	1.398	0.254	1.520	0.217	1.666	0.179
		**Carotenoids**		**Chl** *** b/a***									
		**F**	***P***	**F**	***P***								
Heat	1	24.197	0.000	0.338	0.564								
LR	2	1.864	0.170	1.560	0.224								
LH	2	44.328	0.000	0.007	0.993								
Heat×LR	2	1.702	0.197	4.255	0.022								
Heat×LH	2	4.904	0.013	1.594	0.217								
LR×LH	4	0.372	0.827	0.778	0.547								
Heat×LR×LH	4	0.666	0.620	0.890	0.480								
***GOIs***	**df**	**psbA**		**psbD**		**psbC**		**PSBS**		**FD**		**RBCS**	
**Effect**		**F**	***P***	**F**	***P***	**F**	***P***	**F**	***P***	**F**	***P***	**F**	***P***
Heat	1	3.932	0.055	0.118	0.733	0.860	0.360	33.994	**0.000**	77.251	**0.000**	70.165	**0.000**
LR	2	2.006	0.149	0.269	0.766	0.233	0.793	0.274	0.762	0.276	0.760	1.491	0.231
LH	2	14.872	**0.000**	9.273	**0.001**	1.797	0.180	13.667	**0.000**	0.701	0.503	0.606	0.548
Heat×LR	2	3.042	0.060	0.631	0.538	1.233	0.303	1.271	0.293	1.844	0.173	0.954	0.389
Heat×LH	2	1.694	0.198	2.405	0.105	0.106	0.900	0.439	0.648	0.115	0.892	0.244	0.784
LR×LH	4	0.503	0.733	1.544	0.210	1.028	0.406	0.542	0.706	0.465	0.761	0.563	0.690
Heat×LR×LH	4	2.220	0.086	1.583	0.200	0.836	0.511	0.380	0.822	0.892	0.479	1.037	0.393
		**CAB-151**		**POR**		**HSP90**		**SHSP**		**AOX**		**BI**	
		**F**	***P***	**F**	***P***	**F**	***P***	**F**	***P***	**F**	***P***	**F**	***P***
Heat	1	161.684	**0.000**	59.937	**0.000**	9.036	**0.005**	0.732	0.398	424.021	**0.000**	287.155	**0.000**
LR	2	1.686	0.200	2.199	0.126	1.414	0.256	0.120	0.887	0.909	0.412	1.479	0.241
LH	2	1.007	0.375	2.532	0.094	1.919	0.162	1.108	0.341	16.731	0.000	8.133	**0.001**
Heat×LR	2	1.484	0.240	1.734	0.191	1.166	0.323	0.126	0.882	0.209	0.812	0.635	0.536
Heat×LH	2	0.205	0.816	0.801	0.457	0.087	0.917	0.029	0.972	4.518	**0.018**	5.213	**0.010**
LR×LH	4	0.572	0.685	0.600	0.665	0.823	0.519	0.237	0.915	0.837	0.511	0.626	0.647
Heat×LR×LH	4	1.212	0.323	0.655	0.627	1.377	0.261	0.157	0.959	1.707	0.170	0.401	0.807
* P <0.05 are in bold. Results of SNK pairwise tests are outlined in * **Supplementary Table S3**.													

**Figure 4:**
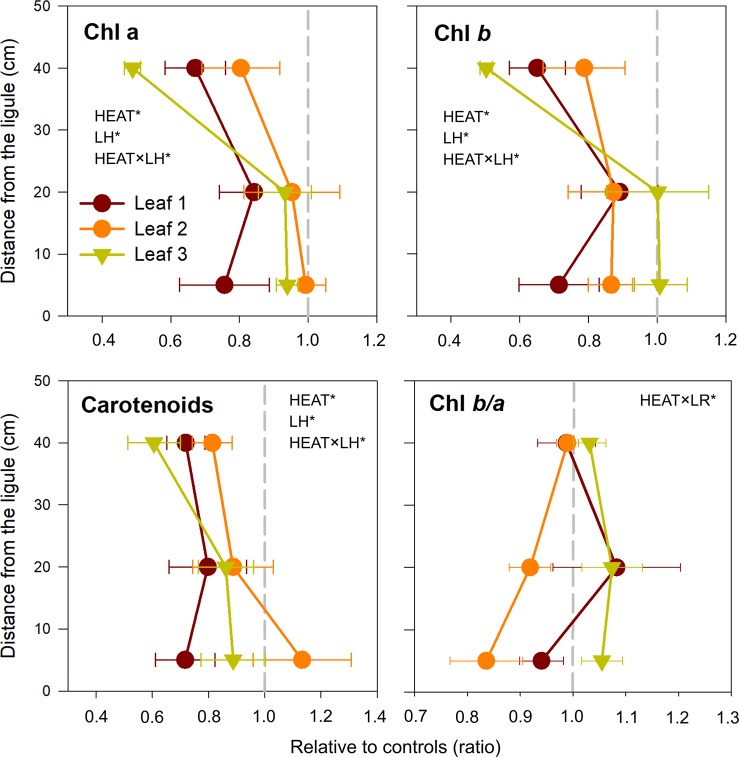
Changes in chlorophyll *a* (Chl *a*), chlorophyll *b* (Chl *b*), total carotenoids concentrations, and Chl *b/a*, in heated relative to control plants (ratio). Values on the left or right of the dashed gray line represent a decrease or increase in respect to controls, respectively. Data are mean ± SE (*n* = 3). Significant results of three-way analyses of variance are reported on the top of the graphs. For details of SNK post-hoc tests see **Table S3.**

Photosynthetic pigment content of *P. oceanica* (Chl*a*, Chl*b*, and carotenoids) was affected by the interaction Heat × LH (**Table 3**). Specifically, both chlorophylls and total carotenoids decreased significantly after heat stress only in uppermost leaf segments (SNK, *P* < 0.001) (**Tables 3** and **S3**; **Figure 4**). On the contrary, the antenna size (Chl *b*/*a*) was similar in control and heated plants, with a small decrease only in leaf 2 (SNK, *P* = 0.09) (**Tables 3** and **S3**; **Figure 4**).

### Target Gene Expression

Three-way PERMANOVA confirmed a strong significant effect of acute heat stress [*P*_(perm)_ < 0.001; **Table 2**] and leaf height [*P*_(perm)_ < 0.05; **Table 2**] on the multivariate gene-expression response of *P. oceanica*, with no apparent interaction between the two factors.

On the other hand, the analysis of individual gene expression by means of univariate three-way ANOVA, revealed that such interaction was restricted to genes involved in respiration and plant PCD (*AOX* and *BI*). Most of targeted genes (9 out of 12 GOIs) were significantly affected by intense warming, regardless the leaf rank and/or height, for example those involved in photosynthesis, light harvesting/chlorophyll biosynthesis and carbon assimilation pathways (**Table 3**).

**Figure 5:**
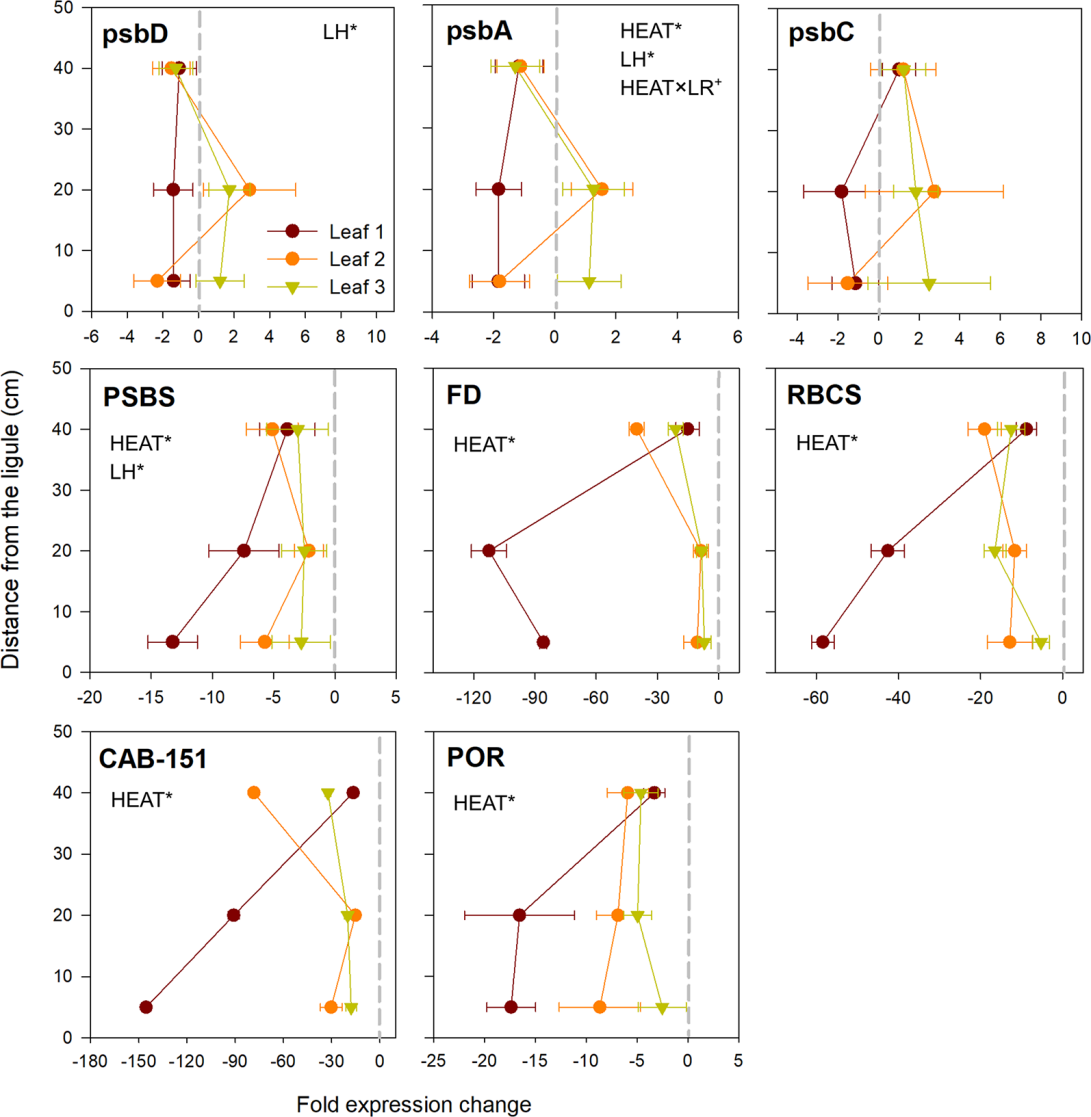
Relative expression of genes involved in photosynthesis, chlorophyll metabolism and carbon fixation in heated *vs.* control plants. Data are mean ± SE (*n* = 3). Negative fold changes represent transcript down-regulation and *vice versa*. Significant results of three-way analyses of variance are reported on the top of the graphs. For details of SNK post-hoc tests see **Table S3.**

Core photosystem II subunits (*psbD* and *psbA*) exhibited a peculiar behavior, with a general pattern of down-regulation in the youngest leaf (rank 1), whereas in the leaves 2 and 3 they were either slightly up or down-regulated depending on the leaf sections considered (**Figure 5**). Only for *psbA*, the response to heat stress varied significantly with leaf rank (SNK, control *vs.* heated in leaf 1: *P* <0.05; **Table S3**).

Genes involved in non-photochemical quenching (*PSBS*), photosynthetic electron transport (*FD*) and Calvin cycle (*RBCS*) were all negatively affected by acute warming. They were significantly down-expressed in all established leaf sections (*P* < 0.001; **Table 3**), with strongest values recorded in youngest leaf tissues (basal and middle portions of leaf 1), where e.g. *RBCS* and *FD* were down-regulated up to 60 and 100 fold changes, respectively, when compared to control plants (**Figure 5**). A quite similar behavior was shown by chlorophyll-related genes (light harvesting and chlorophyll biosynthesis) (*CAB-151* and *POR*), which were significantly negatively affected by heat stress (*P* < 0.001; **Table 3**), again at higher level in basal and middle sections of leaf 1 (**Figure 5**).

**Figure 6:**
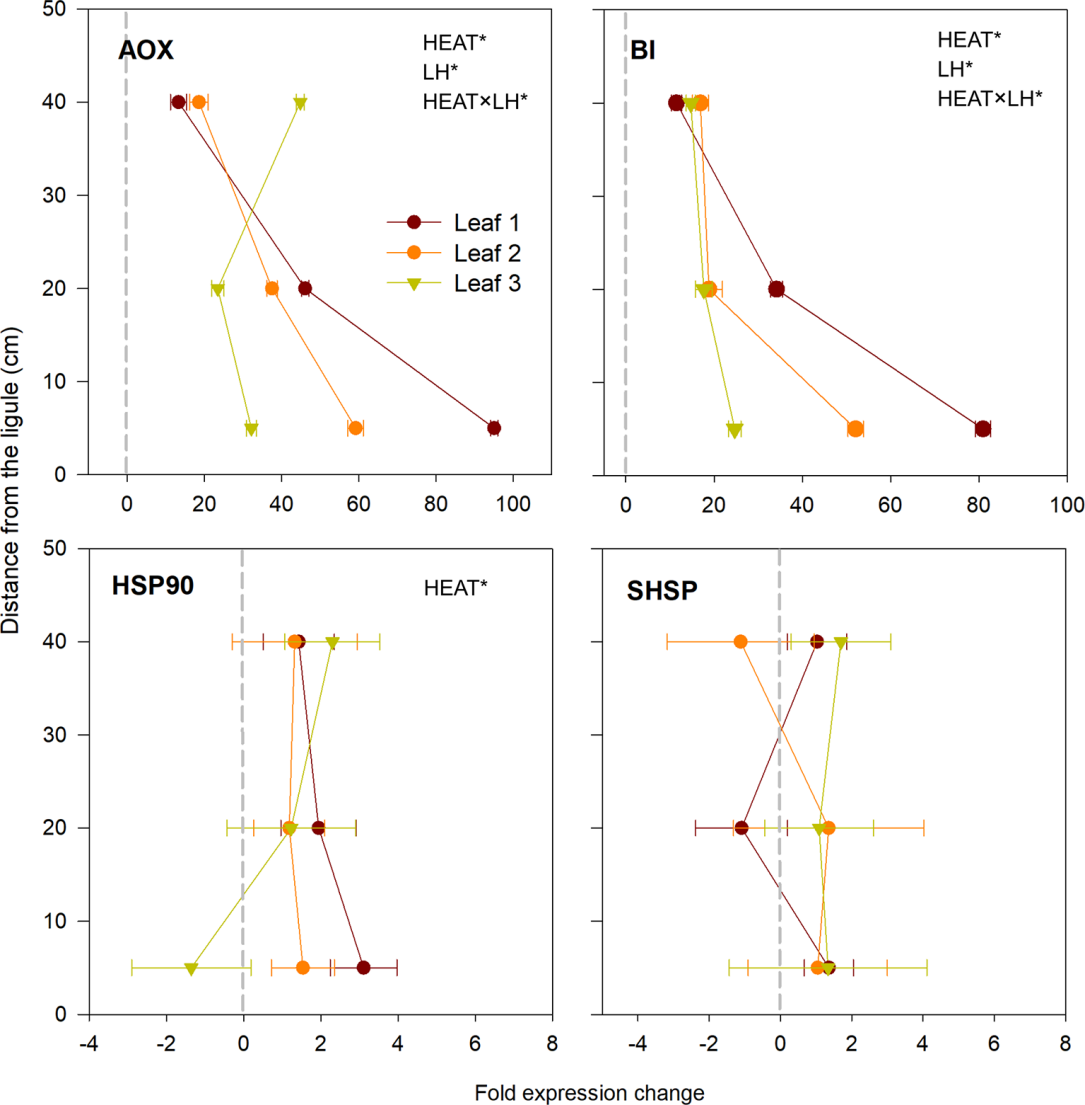
Relative expression of genes involved in respiration, programmed cell death and heat-shock proteins in heated *vs.* control plants. Data are mean ± SE (*n* *=* 3). Negative fold changes represent transcript down-regulation and *vice versa*. Significant results of three-way analyses of variance are reported on the top of the graphs. For details of SNK post-hoc tests see **Table S3.**

Among the two selected HSP proteins (*HSP90* and *SHSP*), only *HSP90* was significantly over-expressed in heated plants with respect to controls (*P* < 0.01; **Table 3**), with highest values in the basal section of leaf 1 and upper portion of leaf 3 (**Figure 6**). *AOX* and *BI* were the two most up-regulated genes under heat stress of the whole gene-expression dataset (*P* < 0.001; **Table 3**). They were significantly induced in all sections selected along the leaf length (SNK, control *vs.* heated in B, M and H: *P* <0.001; **Table S3**), particularly in basal segments of leaves 1 and 2, where they reached expression values up to 90-fold changes higher than control plants (**Figure 6**).

### Global DNA Methylation

**Figure 7:**
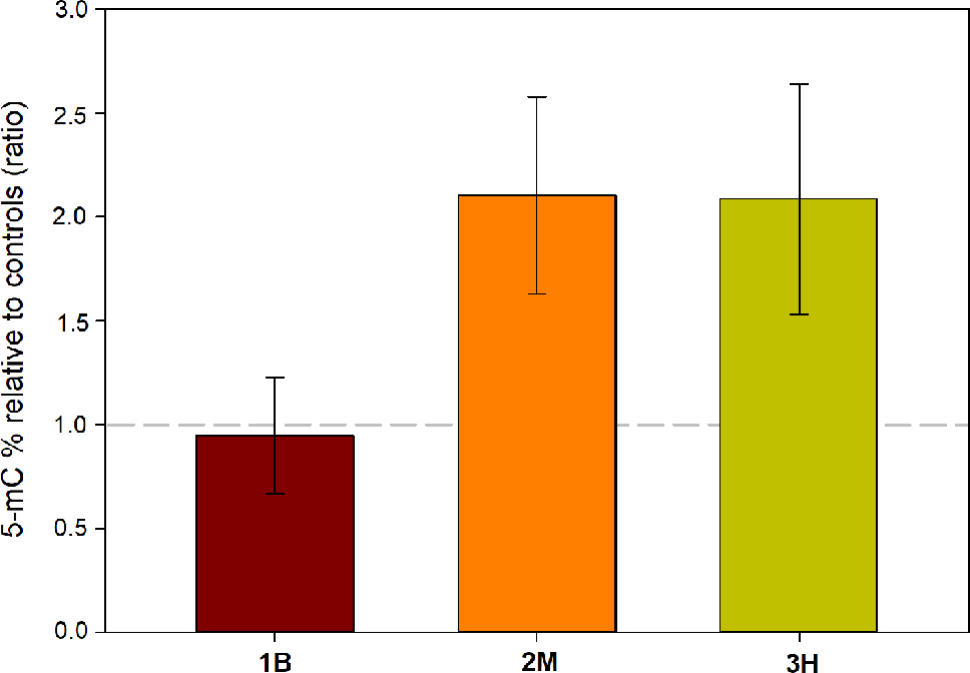
Changes in global DNA methylation level in leaf 1**—**basal (1B), leaf 2**—**medium (2M), and leaf 3**—**high (3H), in heated relative to control plants (ratio). Values above or below the dashed gray line represent an increase or decrease of % 5-mC in respect to controls, respectively. Data are mean ± SE (*n* *=* 3).

Two-way ANOVA highlighted a significant effect of intense warming on global genome methylation (5-mC) (*P* < 0.05), without a significant leaf-section effect (**Table 4**). Only in intermediate (2M) and oldest leaf portions (3M), heat stress-induced increase in % of methylated DNA was evident (Control: 2M = 2.97 ± 0.35, 3H = 4.15 ± 0.80; Heated: 2M = 6.24 ± 2.33, 3H = 8.66 ± 3.62), whereas youngest leaf segments (1B) had a comparable level of methylated DNA in heated and control conditions (Control: 1B = 5.72 ± 1.26; Heated: 1B = 5.41 ± 2.50) (**Figure 7**).

**Table 4:** Results of 2-way analysis of variance to assess the effect of leaf section (LS) and heat stress on global DNA methylation level (5-mC).

**Two-way Analysis of Variance**			
***Global DNA methylation***			
**Effect**	**df**	**F**	**P**
Heat	1	6.176	**0.029**
Leaf section	2	1.0730	0.373
Heat × LS	2	2.065	0.169
*P <0.05 are in bold.*			

## Discussion

Results presented here revealed that photo-physiological and molecular responses of the seagrass *P. oceanica* to short-term acute heat stress vary with tissue age. Leaf-age segments activate variable strategies to withstand heat stress (i.e. thermal plasticity), thus influencing whole plant survival. The vertical age gradient existing along the longitudinal axis of *P. oceanica* leaves, from the base toward the tip, appeared to affect heat-stress responses more than the horizontal age gradient, responsible for leaf rank-differences. In addition, the strong irradiance gradient existing within the complex canopies formed by this species, which is interconnected with the vertical age gradient (Ruocco et al., 2019), plays a role in shaping the observed pattern of responses to warming exposure.

Many chlorophyll fluorescence parameters are commonly used to study the functioning of the photosynthetic apparatus under a range of abiotic stresses (Baker, 2008). In particular, basal fluorescence (F_0_) and photochemical efficiency (Fv/Fm) are photo-physiological tools used as indicators of heat-induced thermal damages to PSII (Yamada et al., 1996), and have been shown to highly correlate with photosynthetic thermo-tolerance (Knight and Ackerly, 2002). Here, the exposure of *P. oceanica* plants to acute heat stress resulted in a great spatial heterogeneity in chlorophyll fluorescence patterns in/across leaves, as often demonstrated in terrestrial plants under diverse abiotic stresses (e.g. drought, osmotic stress, heat; Xu et al., 2011; Sperdouli and Moustakas, 2012; Li et al., 2015) and during leaf senescence (Wingler et al., 2004). Warming exposure caused a progressive loss of photochemical efficiency (Fv/Fm) from the young leaf bases to the distal (old) leaf segments.

Interestingly, such changes were driven by leaf sections-specific variations in either basal (F_0_) or maximum (Fm) chlorophyll fluorescence that could reflect different thermo-tolerance and/or the induction of diverse strategies to cope with warming conditions. Uppermost leaf sections, which had the strongest decline in chlorophyll content, also experienced the strongest down-regulation in Fv/Fm, and this was caused by a reduction in Fm values, while basal fluorescence (F_0_) was comparable to that of control plants. On the contrary, Fv/Fm depression in medium leaf tissues was due to the rise of F_0_, which exhibited the highest values in these sections, combined with a decrease in Fm. Contemporary to Fv/Fm loss, NPQ rose in heated plants, similar to what found in terrestrial species (e.g. Tang et al., 2007; Yin et al., 2010). NPQ harmlessly quenches the excitation of chlorophyll within the light-harvesting antennae of PSII by converting excitation energy into thermal energy (Ruban, 2016). Here, there was a tendency for middle and upper leaf segments to display higher NPQ values, possibly resulting from their long-term exposure to high light levels. This suggests a greater contribution of NPQ when heat stress combines with high light conditions, as the energy input by light could exceed the demand of CO_2_ fixation metabolism, under diminished photosynthesis (Yin et al., 2010). Especially in the oldest analyzed leaf sections, the higher ability for inducing NPQ could protect PSII from heat damage, as evidenced by the lack of adverse effects on F_0_ (PSII inactivation), while Fm decrease reflects the stimulation of photo-protective mechanisms and non-radiative energy loss (Urban et al., 2017). Basal (youngest) leaf sections exhibited the smallest variations in Fv/Fm, as resulting from no F_0_ induction and only little Fm decrease, while they do not activate photo-protective processes (NPQ), being exposed to very low irradiance levels, besides heat stress.

The observed responses at photo-physiological level reflected molecular alterations in terms of differential leaf age-related gene-expression patterns. Acute heat stress caused minor changes in the expression of genes encoding for subunits of the core complex of PSII (although only significant for *psbA*), and major changes in genes for key components of the photosynthetic electron transport (*FD*), Calvin cycle (*RBCS*), light harvesting system (*CAB-151*) and chlorophyll biosynthetic pathway (*POR*). Most of these genes were significantly suppressed under heat stress without any interaction with LH and/or LR; however, the youngest sections of leaf 1 (1B and 1M) were undoubtedly the most negatively affected, as this pattern of down-regulation, although with a variable extent among target genes, was very severe in such tissues. Conversely, leaves 2 and 3 showed a similar behavior and a higher ability to regulate gene expression for buffering/offsetting the down-regulation of key functional genes, as reflected by the lower suppression of PSII components and other photosynthesis-related genes. Under moderate heat stress, the enhancement of electron transport and ATP synthesis, driven by an increased activity of the Calvin cycle, can suppress the accumulation of excess electrons along the chloroplast electron transport chain, therefore lessening the production of ROS and enhancing PSII repair (Hancock Robert et al., 2013; Marín-Guirao et al., 2016; Ueno et al., 2016). On the contrary, photosynthesis inhibition occurs in severely heat-stressed leaves and is generally accompanied by the down-regulation of transcripts and proteins associated with primary carbon assimilation, photochemistry and electron transport (Nouri et al., 2015), as well as from lesser accumulation of light-harvesting pigments due to impaired Chl synthesis, its accelerated degradation or a combination of both (Kumar Tewari and Charan Tripathy, 1998). This could suggest a higher sensitivity to heat stress of immature, developing leaves, and points for the inhibition of the PSII repair cycle, whole CO_2_ assimilation and chlorophyll biosynthesis in such tissues (Mathur et al., 2014; Nishiyama and Murata, 2014).

Another consistent hypothesis is that the observed patterns of gene expression are due to youngest leaf tissues do not undergo further maturation under heat stress, in agreement with the slowdown of the overall leaf growth rate. Young *P. oceanica* leaves, in particular basal and medium segments, are constitutively enriched in transcripts encoding for functional and structural proteins that are required for the assembly of the photosynthetic apparatus, plastids' differentiation and the subsequent acquisition of photosynthetic competence (Ruocco et al., 2019). The maintenance of the transcriptional machinery devoted to growth and development-related processes requires high-energy costs, especially considering that these immature tissues are sink structures, largely dependent on imported carbohydrates from mature and fully photosynthetically-competent leaf tissues (Evert et al., 1996). Heat stress-induced growth arrest would allow the diversion of energy for the activation of protective/stress responses at the expenses of growth (Kosová et al., 2011), letting the plant to preserve these fundamental leaf segments that contribute to whole plant development.

This is corroborated by the higher accumulation of the transcript encoding for *HSP90*, mainly in young leaf segments of *P. oceanica* exposed to 34°C. The massive production of HSPs, chaperons and chaperonins, is generally one of the most important characteristic of thermo-tolerance, as they play a major role in correcting adverse conformational changes resulting in non-functional proteins (protein denaturation) under elevated temperature (Kosová et al., 2011; Qu et al., 2013). In addition, the most up-regulated genes of the entire gene-expression dataset were *AOX* and *BI*, encoding for the mitochondrial Alternative oxidase and Bax Inhibitor-1, respectively. They were significantly over-expressed in all leaf segments selected along *P. oceanica* leaves, with the basal portion of leaf 1 (1B) featuring the highest fold changes in respect to controls. The induction of *AOX* and *BI* under heat stress confirms their pivotal role in mediating seagrass stress acclimation (Marín-Guirao et al., 2017; Tutar et al., 2017; Traboni et al., 2018). Aox pathway minimizes heat shock-mediated ROS production across the mitochondrial electron-transport chain (Vanlerberghe, 2013), while Bax Inhibitor acts preventing ROS-induced programmed cell death (Watanabe and Lam, 2006). Notably, Aox represents a link between metabolic activities and signaling, as it mediates the creation of a retrograde signaling network from the mitochondrion to the nucleus for regulating stress-related gene expression (Saha et al., 2016), as well it is involved in the induction of NPQ in the chloroplast, which is coherent with our observations (Vishwakarma et al., 2015). The simultaneous induction of *HSP90*, *AOX* and *BI* is coherent with the "protective" strategies that could be adopted by immature leaf tissues, as discussed above. Following this view, under heat stress, energy and resources would be devoted to trigger a protective/conservative response, aimed at preserving the integrity of youngest leaf tissues, those fundamental for plant survival after the stress cessation, and at inhibiting ROS accumulation and subsequent induction of programmed-cell death in such tissues (Kosová et al., 2011).

Observed patterns of gene expression under heat stress could be at least partially regulated by heat-stress induced epigenetic changes. Global DNA methylation analysis showed that % 5-mC only increased in the intermediate (2M) and oldest analyzed leaf tissues (3H) under warming conditions, while basal sections (1B) showed values comparable to controls. We did not have a precise explanation for such intra-organ variations; however, our results confirm the involvement of the epigenetic machinery in the modulation of *P. oceanica* heat acclimation (Marín-Guirao et al., 2017; Marín-Guirao et al., 2019), as already demonstrated in terrestrial higher plants (e.g. Liu et al., 2015). Epigenetic modifications, including DNA methylation, histone modifications/variants and small/long non-coding RNAs, can regulate the expression of heat-responsive genes and function to prevent heat-related damages (Liu et al., 2015). At whole genome level, DNA methylation has been shown to be differently influenced by heat stress in different species. For example, in *Arabidopsis* and cork oak, heat exposure resulted in an increased global DNA methylation (Boyko et al., 2010; Correia et al., 2013), resembling results presented here for *P. oceanica*. However, it appears that there is no consistent trend in total DNA methylation changes under heat stress, since genome-wide hypomethylation has also been shown (Liu et al., 2015). Intriguingly, two previous studies in *P. oceanica* demonstrated whole genome hypermethylation in response to light-limitation stress and cadmium exposure (Greco et al., 2012; Greco et al., 2013), besides methylation changes at specific loci and the up-regulation of a DNA chromomethylase involved in both maintenance and *de novo* DNA methylation. Thus, increasing whole DNA methylation level might be a recurring response in *P. oceanica* during stress acclimation. Two main hypothesis on its significance can be proposed: i) higher DNA methylation could suppress retrotransposition, which is triggered by environmental stressors, thus it serves to preserve genome stability (Mirouze and Paszkowski, 2011); ii) increase in DNA methylation may down-regulate the expression of the transcriptome slowing down overall metabolism, which would allow the plants to conserve energy needed to overcome the temporary challenge (Saraswat et al., 2017). Nonetheless, the cause-effect correlation between temperature and DNA methylation remains uncertain, as detailed analyses are hampered by the lack of genomic information in this species. First evidence of the relationships between intragenic methylation and flexible expression of specific genes across latitudinal gradients and under warming conditions in seagrasses, come from *in silico* transcriptome analysis of normalized CpG content (CpG_O/E_ ratio) (Entrambasaguas et al.,submitted).

**Figure 8:**
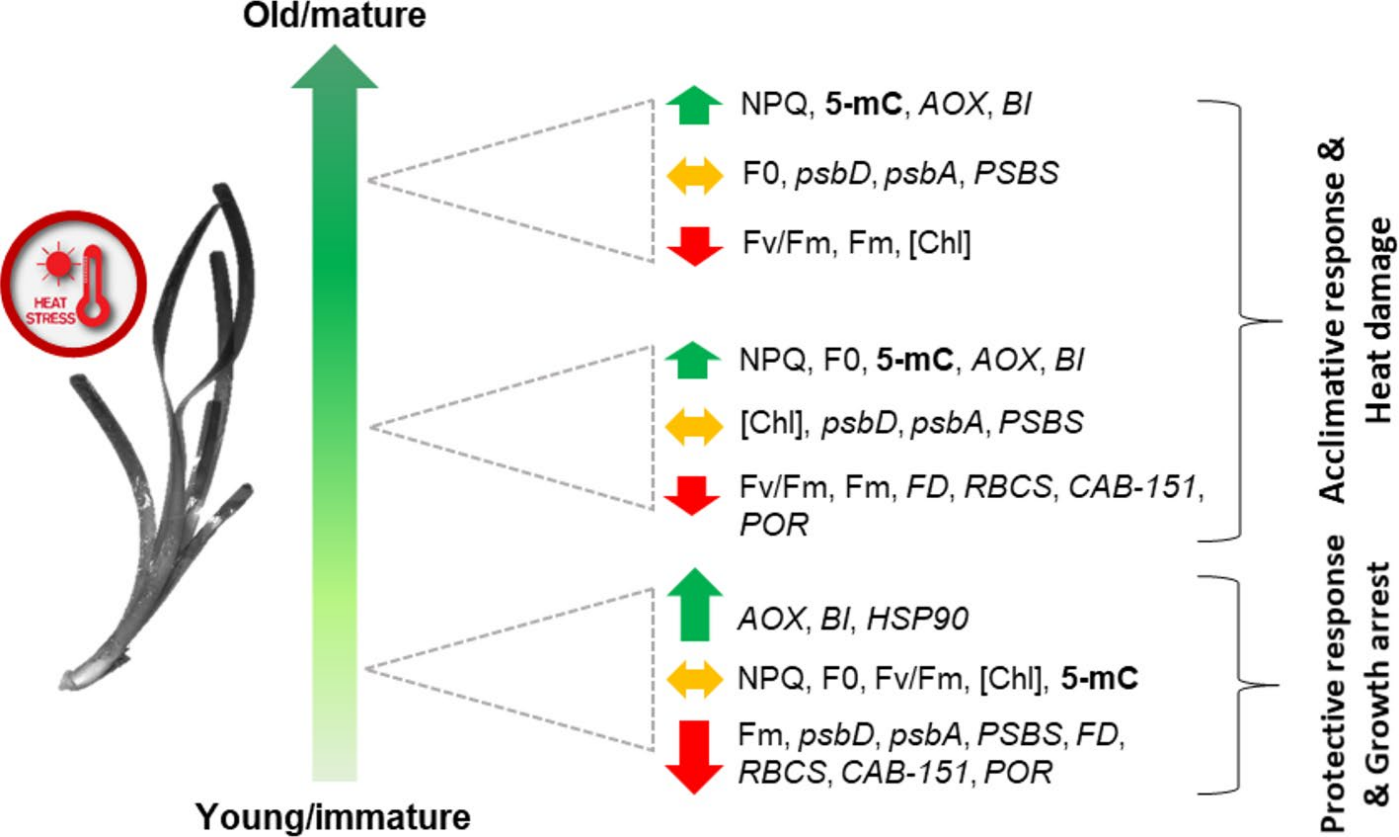
Schematic (qualitative) representation of main leaf-age related responses of *P. oceanica* to acute heat stress. Combined responses of both leaf-age gradients (vertical and horizontal) are depicted. Total methylation (5-mC) is in bold; photo-physiological parameters (NPQ, Fv/Fm, F_0_, Fm, [Chl]) are in regular letters; genes (*psbD, psbA, PSBS, FD, RBCS, CAB-151, POR, AOX, BI,* and *HSP90*) are in italics. The length of the arrows reflects the intensity of gene up or down-regulation.

In conclusion, these data revealed, for the first time, the presence of differential leaf age-dependent stress-induced epigenetic and gene-expression changes in *P. oceanica*, underlying photo-physiological and growth responses to heat stress (**Figure 8**). A high heat-induced heterogeneity in chlorophyll fluorescence parameters was detected in/across *P. oceanica* leaves, which suggest a differential thermal-plasticity of leaf-age segments. Young/immature leaf sections, those fundamental for the overall shoot growth, showed a dramatic down-regulation of key genes involved in photosynthetic electron transport, carbon assimilation and Chl biosynthesis, concurrently with the extreme over-expression of genes involved in alternative mitochondrial respiration and PCD suppression, which points for the investment in protective responses and growth/maturation arrest. On the other hand, mature leaf tissues displayed a more acclimative response, since they were able to offset extreme gene down-regulation, possibly also through the involvement of DNA methylation changes, which appear to be triggered only in these tissues. The biological significance of epigenetic changes identified through this study deserves much further investigation aimed at identifying their targets, as well as their location within the genetic sequences; however, the re-patterning of genome methylation seems to be a key regulator of stress acclimation processes in *P. oceanica,* and perhaps in other seagrass species. Importantly, the status of genome methylation could also represent a useful bio-monitoring tool for the early detection of stress in seagrasses (Greco et al., 2013). In light of both gene-expression and epigenetic results, future studies should be designed to test the presence of a thermo-memory (i.e. the maintenance of acquired thermo-tolerance in the absence of stress) in seagrasses, as it has been proposed that possible mechanisms underlying this process are the accumulation of proteins, transcription factors or protective metabolites, as well as epigenetic modifications, which can last for generations (Walter et al., 2013). In particular, the control of HSP (or other chaperones) abundance has been recently demonstrated to be a key mechanism for determining the duration of the thermo-memory phase in *Arabidopsis* (Sedaghatmehr et al., 2016).

Our results also have fundamental methodological implications when assessing stress-induced effects on physiological and molecular properties in seagrasses. Intermediate sections of rank leaves 2 and 3 are generally considered the most representative of the whole-shoot metabolic state, since they are of key importance as energy source (Alcoverro et al., 1998; Enríquez et al., 2002; Olivé et al., 2013). Here, we demonstrated that short-term acute heat stress dramatically affected young leaf tissues, especially in terms of differential gene-expression patterns. Therefore, molecular/physiological evaluations conducted only on adult tissues, as common practice in seagrass research, could give inadequate estimates of the overall plant state, and should not be considered as a proxy for the whole shoot. In general, sampling a range of leaf-age classes would yield the most representative tolerance measurements under abiotic stress. In alternative, the extreme heterogeneity of leaf-related responses, would suggest the use of other plant organs, such as apical meristems, as proxies of seagrass stress status in future research.

## Data Availability Statement

All datasets generated for this study are included in the article/**Supplementary Material**.

## Author Contributions

MR, GP, and LM-G conceived the experiment. MR, GP, and LM-G participated in the mesocosm maintenance. MR performed the molecular analyses with the assistance of PL. MR and LM-G performed photo-physiological and morphological analyses. MR wrote the manuscript with the help of all other co-authors.

## Funding

The building of the SZN mesocosm system was supported by the PON BIOforIU (PONa3_00025, MIUR). Open access publication fee was partially covered by Stazione Zoologica Anton Dohrn.

## Conflict of Interest

The authors declare that the research was conducted in the absence of any commercial or financial relationships that could be construed as a potential conflict of interest.
