# Supplementary material for: Differential Leaf Age-Dependent Thermal Plasticity in the Keystone Seagrass Posidonia oceanica
Source: Front Plant Sci. 2019 Nov 29;10:1556. doi: 10.3389/fpls.2019.01556 (PMC6900526; doi:10.3389/fpls.2019.01556)
Supplement: Supplementary file 1 [file DataSheet_1.docx]

**Differential Leaf Age-Dependent Thermal Plasticity in the Keystone Seagrass *Posidonia oceanica***

Miriam Ruocco^1^, Pasquale de Luca^1^, Lazaro Marín-Guirao^1,2^ & Gabriele Procaccini^1^

^1^Stazione Zoologica Anton Dohrn, Villa Comunale, 80121 Naples, Italy

^2^ Seagrass Ecology Group, Oceanographic Center of Murcia, Spanish Institute of Oceanography, C/ Varadero, 30740 San Pedro del Pinatar, Spain.

**Table S1**. Scores of the 11 microsatellite markers used for genotyping the *P. oceanica* ramets used in this study

| *Samples*/*SSR* | Po-15* | | Po-5-10* | | Po-4-3* | | Po5-39* | | Poc-42** | | Poc-45** | | Po-5* | | Poc-5** | | Poc-35** | | Poc-trn** | | Poc-26** | |
| --- | --- | --- | --- | --- | --- | --- | --- | --- | --- | --- | --- | --- | --- | --- | --- | --- | --- | --- | --- | --- | --- | --- |
| **C1^+^** | 142 | 142 | 161 | 163 | 164 | 166 | 177 | 177 | 0 | 0 | 140 | 140 | 161 | 179 | 170 | 173 | 196 | 196 | 301 | 301 | 316 | 316 |
| **C2^+^** | 144 | 158 | 161 | 161 | 164 | 168 | 175 | 175 | 0 | 0 | 117 | 140 | 179 | 193 | 170 | 170 | 190 | 196 | 301 | 301 | 316 | 316 |
| **C3^+^** | 144 | 158 | 161 | 161 | 164 | 168 | 175 | 177 | 210 | 216 | 117 | 140 | 179 | 193 | 170 | 170 | 190 | 196 | 301 | 301 | 316 | 316 |
| **C4^+^** | 132 | 144 | 161 | 163 | 164 | 166 | 175 | 177 | 210 | 216 | 117 | 140 | 161 | 179 | 170 | 170 | 190 | 190 | 301 | 301 | 316 | 316 |
| **C5^+^** | 132 | 144 | 161 | 165 | 164 | 164 | 177 | 177 | 210 | 216 | 117 | 140 | 179 | 193 | 170 | 173 | 196 | 196 | 301 | 301 | 316 | 316 |
| **C6^+^** | 132 | 144 | 161 | 165 | 166 | 166 | 177 | 177 | 210 | 210 | 117 | 140 | 179 | 189 | 170 | 170 | 190 | 196 | 301 | 301 | 316 | 316 |
| **T1** | 142 | 144 | 161 | 163 | 164 | 164 | 175 | 177 | 216 | 216 | 117 | 117 | 179 | 193 | 170 | 170 | 196 | 196 | 301 | 301 | 316 | 316 |
| **T2** | 142 | 144 | 161 | 163 | 164 | 164 | 175 | 177 | 216 | 216 | 117 | 117 | 179 | 193 | 170 | 170 | 196 | 196 | 301 | 301 | 316 | 316 |
| **T3** | 132 | 142 | 161 | 165 | 164 | 164 | 177 | 177 | 210 | 216 | 117 | 140 | 179 | 193 | 170 | 173 | 196 | 196 | 301 | 301 | 316 | 316 |
| **T4** | 132 | 144 | 157 | 165 | 164 | 164 | 175 | 177 | 210 | 216 | 117 | 140 | 161 | 179 | 170 | 170 | 196 | 196 | 301 | 301 | 316 | 316 |
| **T5** | 132 | 144 | 157 | 165 | 164 | 164 | 175 | 177 | 210 | 216 | 117 | 140 | 161 | 179 | 170 | 170 | 196 | 196 | 301 | 301 | 316 | 316 |
| **T6** | 132 | 142 | 161 | 165 | 164 | 164 | 175 | 177 | 210 | 216 | 117 | 140 | 179 | 193 | 0 | 0 | 196 | 196 | 301 | 301 | 316 | 316 |

**^+^** Data from Ruocco et al., 2019

*Alberto et al. 2003; ** Procaccini and Waycott, 1998

Ruocco M, Marín-Guirao L, Procaccini G (2019) Within-and among-leaf variations in photo-physiological functions, gene expression and DNA methylation patterns in the large-sized seagrass *Posidonia oceanica*. Marine Biology 166(3): 24

Alberto F, Correia L, Arnaud-Haond S, Billot C, Duarte CM, Serrão E (2003) New microsatellite markers for the endemic Mediterranean seagrass *Posidonia oceanica*. Molecular Ecology Notes 3: 253-255

Procaccini G, Waycott M (1998) Microsatellite loci identified in the seagrass *Posidonia oceanica* (L.) Delile. Journal of Heredity 89: 562-568

**Table S2.** Photosynthetic parameters and pigment concentrations determined in B, M and H sections of leaves 1, 2 and 3, under control (C) and heated (H) conditions. R-ETR (μmol electrons m^-2^ s^-1^); Chl *a*, Chl *b* and total carotenoids (µg cm^-1^); Chl *b/a* (molar ratio). Values are means (SE) for *n*=3

|  | **F_0_** | | **Fm** | | **Fv/Fm** | | **ΔF/Fm'** | | **r-ETR** | | **NPQ** | | **Chl *a*** | | **Chl *b*** | |
| --- | --- | --- | --- | --- | --- | --- | --- | --- | --- | --- | --- | --- | --- | --- | --- | --- |
|  | C | H | C | H | C | H | C | H | C | H | C | H | C | H | C | H |
| *Leaf 1* | | | | | | | | | | | | | | | | |
| B | 332.33  (12.50) | 347.17  (19.44) | 1431  (54.26) | 1013  (63.47) | 0.77  (0.00) | 0.65  (0.02) | 0.71  (0.03) | 0.63  (0.03) | 15.77  (2.07) | 9.87  (0.99) | 0.50  (0.16) | 1.00  (0.08) | 21.48  (3.52) | 16.25  (0.91) | 10.62  (1.64) | 7.58  (0.40) |
| M | 343.50  (3.97) | 411.50  (26.70) | 1466  (11.25) | 1053  (39.26) | 0.77  (0.00) | 0.61  (0.03) | 0.69  (0.01) | 0.49  (0.01) | 23.47  (2.21) | 12.88  (0.90) | 0.69  (0.05) | 1.58  (0.18) | 30.75  (1.89) | 25.90  (2.98) | 14.86  (1.18) | 13.24  (1.28) |
| H | 380.67  (10.89) | 373.17  (40.42) | 1469  (7.75) | 822  (143.76) | 0.74  (0.01) | 0.52  (0.03) | 0.68  (0.01) | 0.41  (0.03) | 23.12  (1.36) | 11.80  (0.85) | 1.39  (0.21) | 3.46  (0.53) | 34.27  (2.34) | 23.00  (2.86) | 17.01  (1.26) | 11.07  (1.12) |
| *Leaf 2* | | | | | | | | | | | | | | | | |
| B | 372.50  (8.54) | 403.17  (17.45) | 1609  (28.70) | 1296  (94.18) | 0.77  (0.00) | 0.68  (0.04) | 0.74  (0.04) | 0.65  (0.04) | 16.20  (1.66) | 11.58  (2.05) | 0.51  (0.08) | 0.83  (0.06) | 20.04  (0.81) | 19.88  (1.08) | 10.78  (0.47) | 9.34  (0.59) |
| M | 365.50  (2.75) | 471.33  (6.98) | 1575  (4.07) | 1287  (92.68) | 0.77  (0.00) | 0.63  (0.03) | 0.73  (0.01) | 0.53  (0.05) | 23.03  (0.48) | 12.90  (1.60) | 0.64  (0.04) | 1.38  (0.23) | 29.84  (2.40) | 28.43  (3.94) | 15.11  (1.14) | 13.19  (1.73) |
| H | 372.00  (4.44) | 412.40  (9.22) | 1483  (14.27) | 936  (43.07) | 0.75  (0.00) | 0.53  (0.02) | 0.68  (0.02) | 0.36  (0.03) | 24.25  (0.84) | 11.33  (1.26) | 0.94  (0.13) | 3.82  (0.54) | 33.80  (2.46) | 27.18  (3.68) | 17.01  (1.27) | 13.40  (1.76) |
| *Leaf 3* | | | | | | | | | | | | | | | | |
| B | 389.83  (8.09) | 512.67  (42.59) | 1593  (50.20) | 1361  (64.62) | 0.76  (0.00) | 0.61  (0.05) | 0.73  (0.01) | 0.55  (0.04) | 16.82  (2.20) | 9.87  (1.27) | 0.60  (0.08) | 0.94  (0.26) | 21.17  (1.21) | 19.88  (0.16) | 10.47  (0.58) | 10.55  (0.60) |
| M | 423.50  (10.69) | 482.00  (15.45) | 1641  (8.98) | 1220  (54.18) | 0.74  (0.01) | 0.60  (0.01) | 0.70  (0.01) | 0.45  (0.03) | 20.00  (0.81) | 12.97  (1.08) | 0.85  (0.05) | 2.67  (0.34) | 28.45  (3.08) | 26.52  (1.43) | 14.26  (1.70) | 14.28  (1.27) |
| H | 419.50  (32.00) | 413.00  (13.80) | 1461  (15.46) | 949  (115.93) | 0.71  (0.03) | 0.55  (0.05) | 0.64  (0.05) | 0.31  (0.07) | 22.09  (1.83) | 9.73  (3.14) | 1.78  (0.32) | 4.65  (1.15) | 34.44  (1.32) | 16.84  (0.72) | 17.39  (0.46) | 8.73  (0.21) |

**Table S2** *(continued)*

|  | **Carotenoids** | | **Chl *b/a*** | |
| --- | --- | --- | --- | --- |
|  | C | H | C | H |
| *Leaf 1* | | | | |
| B | 5.97  (0.84) | 4.28  (0.18) | 0.49  (0.02) | 0.46  (0.00) |
| M | 8.44  (0.65) | 6.73  (1.04) | 0.48  (0.01) | 0.51  (0.06) |
| H | 9.99  (0.72) | 7.18  (0.43) | 0.49  (0.01) | 0.48  (0.03) |
| *Leaf 2* | | | | |
| B | 4.91  (0.40) | 5.57  (0.73) | 0.51  (0.04) | 0.46  (0.01) |
| M | 8.94  (0.98) | 7.94  (0.95) | 0.50  (0.02) | 0.46  (0.01) |
| H | 10.13  (0.67) | 8.24  (0.47) | 0.50  (0.01) | 0.49  (0.00) |
| *Leaf 3* | | | | |
| B | 5.35  (0.37) | 4.75  (0.51) | 0.49  (0.00) | 0.51  (0.02) |
| M | 7.64  (0.85) | 6.58  (0.17) | 0.49  (0.01) | 0.53  (0.03) |
| H | 10.56  (0.48) | 6.40  (0.94) | 0.50  (0.01) | 0.51  (0.01) |

**Table S3.** Results of SNK pairwise tests following 3-way ANOVAs on photo-physiological variables (photosynthetic parameters and pigment content) and GOIs. (***) *P* <0.001, (**) *P* <0.01, (*) *P* <0.05

| **Three-way ANOVA** |  | *SNK pairwise tests* |
| --- | --- | --- |
| *Photo-physiology* | | |
| **F_0_** |  |  |
| Heat*** |  |  |
| LR*** |  | 1 ≠ 2 ≠ 3 |
| LH (*P* =0.06) |  |  |
| Heat×LR |  |  |
| Heat×LH* |  | B: Control ≠ Heated; M: Control ≠ Heated; H: Control = Heated |
| LR×LH |  |  |
| Heat×LR×LH |  |  |
| **Fv/Fm** |  |  |
| Heat*** |  |  |
| LR (*P* =0.06) |  |  |
| LH*** |  | B ≠ M = H |
| Heat×LR |  |  |
| Heat×LH |  |  |
| LR×LH |  |  |
| Heat×LR×LH |  |  |
| **Fm** |  |  |
| Heat*** |  |  |
| LR*** |  | 1 ≠ 2 = 3 |
| LH*** |  | B = M ≠ H |
| Heat×LR |  |  |
| Heat×LH** |  | B: Control ≠ Heated; M: Control ≠ Heated; H: Control ≠ Heated |
| LR×LH |  |  |
| Heat×LR×LH |  |  |
| **ΔF/Fm'** |  |  |
| Heat*** |  |  |
| LR* |  | 1 = 2 ≠ 3 |
| LH*** |  | B ≠ M ≠ H |
| Heat×LR |  |  |
| Heat×LH*** |  | B: Control ≠ Heated; M: Control ≠ Heated; H: Control ≠ Heated |
| LR×LH |  |  |
| Heat×LR×LH |  |  |
| **r-ETR** |  |  |
| Heat*** |  |  |
| LR |  |  |
| LH** |  | B = M ≠ H |
| Heat×LR |  |  |
| Heat×LH* |  | B: Control ≠ Heated; M: Control ≠ Heated; H: Control ≠ Heated |
| LR×LH |  |  |
| Heat×LR×LH |  |  |
| **NPQ** |  |  |
| Heat*** |  |  |
| LR** |  | 1 = 2 ≠ 3 |
| LH*** |  | B ≠ M ≠ H |
| Heat×LR |  |  |
| Heat×LH |  |  |
| LR×LH |  |  |
| Heat×LR×LH |  |  |
| **Chl *a*** |  |  |
| Heat*** |  |  |
| LR |  |  |
| LH*** |  | B ≠ M = H |
| Heat×LR |  |  |
| Heat×LH** |  | B: Control = Heated; M: Control = Heated; H: Control ≠ Heated |
| LR×LH |  |  |
| Heat×LR×LH |  |  |
| **Chl *b*** |  |  |
| Heat*** |  |  |
| LR |  |  |
| LH*** |  | B ≠ M = H |
| Heat×LR |  |  |
| Heat×LH** |  | B: Control = Heated; M: Control = Heated; H: Control ≠ Heated |
| LR×LH |  |  |
| Heat×LR×LH |  |  |
| **Carotenoids** |  |  |
| Heat*** |  |  |
| LR |  |  |
| LH*** |  | B ≠ M ≠ H |
| Heat×LR |  |  |
| Heat×LH* |  | B: Control = Heated; M: Control = Heated; H: Control ≠ Heated |
| LR×LH |  |  |
| Heat×LR×LH |  |  |
| **Chl *b/a*** |  |  |
| Heat |  |  |
| LR |  |  |
| LH |  |  |
| Heat×LR* |  | 1: Control = Heated; 2: Control ≠ Heated (*P* =0.09); 3: Control = Heated |
| Heat×LH |  |  |
| LR×LH |  |  |
| Heat×LR×LH |  |  |
| *GOIs* | | |
| **psbA** |  |  |
| Heat (*P* =0.05) |  |  |
| LR |  |  |
| LH*** |  | B ≠ M = H |
| Heat×LR (*P* =0.06) |  | 1: Control ≠ Heated; 2: Control = Heated; 3: Control = Heated |
| Heat×LH |  |  |
| LR×LH |  |  |
| Heat×LR×LH |  |  |
| **psbD** |  |  |
| Heat |  |  |
| LR |  |  |
| LH** |  | B ≠ M = H |
| Heat×LR |  |  |
| Heat×LH |  |  |
| LR×LH |  |  |
| Heat×LR×LH |  |  |
| **PSBS** |  |  |
| Heat*** |  |  |
| LR |  |  |
| LH*** |  | B = M ≠ H |
| Heat×LR |  |  |
| Heat×LH |  |  |
| LR×LH |  |  |
| Heat×LR×LH |  |  |
| **AOX** |  |  |
| Heat*** |  |  |
| LR |  |  |
| LH*** |  | B ≠ M ≠ H |
| Heat×LR |  |  |
| Heat×LH* |  | B: Control ≠ Heated; M: Control ≠ Heated; H: Control ≠ Heated |
| LR×LH |  |  |
| Heat×LR×LH |  |  |
| **BI** |  |  |
| Heat*** |  | B = M ≠ H |
| LR |  |  |
| LH** |  |  |
| Heat×LR |  |  |
| Heat×LH* |  | B: Control ≠ Heated; M: Control ≠ Heated; H: Control ≠ Heated |
| LR×LH |  |  |
| Heat×LR×LH |  |  |
